# Influence of Temperature on Selected Life-History Traits of Black Soldier Fly (*Hermetia illucens*) Reared on Two Common Urban Organic Waste Streams in Kenya

**DOI:** 10.3390/ani9030079

**Published:** 2019-03-02

**Authors:** Marwa Shumo, Fathiya M. Khamis, Chrysantus M. Tanga, Komi K. M. Fiaboe, Sevgan Subramanian, Sunday Ekesi, Arnold van Huis, Christian Borgemeister

**Affiliations:** 1 Center for Development Research (ZEF), Department of Ecology and Natural Resources Management, 53113 Bonn, Germany; cb@uni-bonn.de; 2 International Centre of Insect Physiology and Ecology (*icipe*), Plant Health Unit, Nairobi 00100, Kenya; fkhamis@icipe.org (F.M.K.); ctanga@icipe.org (C.M.T.); K.Fiaboe@cgiar.org (K.K.M.F.); ssubramania@icipe.org (S.S.); sekesi@icipe.org (S.E.); 3 IPM Department, The International Institute of Tropical Agriculture (IITA), B.P. 2008 (Messa), Nkolbisson, Yaoundé, Cameroon; 4 Department of Plant Sciences, Laboratory of Entomology, Wageningen University & Research, 6700AA Wageningen, The Netherlands; arnold.vanhuis@wur.nl

**Keywords:** organic waste bioconversion, black soldier fly (BSF), rearing temperature, development, growth, longevity, fecundity

## Abstract

**Simple Summary:**

Rapid population growth and urbanization, continued economic growth, shifts in dietary patterns towards more animal source foods are major challenges that sub-Saharan Africa is currently facing. These challenges exert a high demand on agricultural production. Insect species such as the black soldier fly (*Hermetia illucens*) have been identified as potential alternatives for the traditional protein sources used in livestock feed due to their rich nutrient content and the fact that they can be reared on organic side streams. However, black soldier fly larvae are very sensitive to external environments such as temperature and rearing medium. Currently, little is known about the combined influence of temperature and organic waste streams that are readily available in the urban environments of sub-Saharan Africa. Therefore, the aim of this study was to investigate the influence of temperature and organic waste streams on the development of black soldier fly larvae reared on two different organic substrates, i.e., brewers’ spent grain and cow dung. The results show that black soldier fly larvae reared on brewers’ spent grain were more efficient and tolerated a wider range of temperatures in comparison with those reared on cow dung.

**Abstract:**

In sub-Saharan Africa, urban populations are projected to increase by 115% in the coming 15 years. In addition, economic growth and dietary shifts towards animal source foods have put high pressure and demand on agricultural production. The high ecological footprint of meat and dairy production, as well as high feed costs, prevent the livestock sector from meeting the increasing demand in a sustainable manner. Insects such as the black soldier fly (BSF) have been identified as potential alternatives to the conventionally used protein sources in livestock feed due to their rich nutrient content and the fact that they can be reared on organic side streams. Substrates derived from organic byproducts are suitable for industrial large-scale production of insect meal. Although efficient in waste management and in feed production, BSF larvae are very sensitive to the external environment such as temperature and rearing medium. Therefore, we studied the effect of temperature and substrate type, i.e., brewers’ spent grain (SG) and cow dung (CD), on the development and survival of BSF larvae. Both organic substrates were readily available in Nairobi, Kenya, the location of the experiments. In our experiment, 100 3–5-day-old BSF larvae were placed into containers that contained either SG or CD and further treated at temperatures of 15 °C, 20 °C, 25 °C, 30 °C, and 35 °C. The duration of larval development was recorded, and the prepupae were removed, weighed, and placed individually in separate, labeled, 35-mL plastic cups filled with moist sawdust. After emergence, 10 2-day-old adults (5 males and 5 females) from every replica per substrate were transferred into a cage (40 × 40 × 40 cm) and allowed to mate for 24 h at their respective temperatures. The laid egg batches were collected and counted, and the adult flies’ longevity was recorded. The data were subjected to a two-way analysis of variance (ANOVA) using the general linear model procedure. BSF larvae reared on SG developed faster than those reared on CD; the former also favored higher temperatures for their larval development and emergence into adults. The optimum range was 25–30 °C. With increasing temperatures, the longevity of adult BSF decreased, while the fecundity of females increased. Thus, it is possible to take advantage of the readily available SG waste streams in the urban environments of Kenya to produce BSF larvae-derived livestock feed within a short duration of time and at relatively high temperatures.

## 1. Introduction

In 2014, 54% of the world’s population resided in urban areas, while in 1950, this number only constituted 30%; by 2050, two-thirds of the world’s population will live in urban areas [1]. In particular, urban populations in sub-Saharan Africa (SSA) are projected to increase by 115% from today’s figures, from 170 to 360 million, in the next 15 years [1]. As a result, it has been estimated that the global food supply will need to increase by 60% in order to meet the demand of the global population, which is expected to reach 10 billion people by 2050 [2]. Rapid urbanization and the growing human population are coupled with continued economic growth, as well as shifts in dietary preferences towards favoring more animal source foods (ASFs) [3,4,5,6]. Therefore, it is not surprising that both the production and consumption of ASFs in the developing world are forecasted to increase sharply [2]. However, this increase represents a major challenge due to the high ecological footprint associated with the production of meat and dairy products [7,8,9,10,11]. In addition, the level of productivity of many agricultural systems in the developing world is still quite low in terms of the efficiency of land and water resource use [12]. On the other hand, the level of malnutrition associated with insufficient protein consumption in developing countries is still very high [13,14,15,16,17]. Moreover, the costs of livestock production, such as poultry farming, in the developing world are increasing mainly because of the high feed costs, now more than 70% of the production costs [18,19,20]. The use of food ingredients in livestock feed production that are also directly consumed by humans, such as fish and soybean, create a food–feed competition, leading to further increases in ingredient costs and consequently to higher feed costs [19]. Moreover, the massive expansion of soybean cultivation has put pressure on land availability, especially in the tropics, often leading to deforestation and other negative effects for the environment [21]. Therefore, access to affordable feed is significant for more profitable and affordable poultry production.

The current combination of inefficient production and unsustainable consumption patterns points to the need to adopt cost effective production systems, in which alternative protein sources for animal feed with lower ecological footprints are used in order to achieve more sustainable agricultural production and improved food security while safeguarding the already fragile ecosystems and natural resources in the developing world [22,23]. Mass-produced insects have emerged as some of the promising alternatives, as some species can be reared on various types of organic waste, including poultry, pig, and cattle manure, as well as on coffee bean pulp, vegetable residues, catering waste, municipal organic waste, straw, dried distillers’ grains with solubles (DDGS), and fish offal [24,25,26,27]. Among the insect species identified as alternative ingredients for animal feed are the black soldier fly (BSF) *Hermetia illucens* L. (Diptera: Stratiomyidae), the common house fly *Musca domestica* L. (Diptera: Muscidae), and the yellow mealworm *Tenebrio molitor* L. (Coleoptera: Tenebrionidae) [28,29,30,31]. In addition, insects contain high amounts of energy, fatty acids, micronutrients, and especially proteins [32,33,34]. For instance, BSF larvae, which have been used as an accepted feed ingredient for poultry, pigs, and a number of fish and shrimp species, contain about 35–49% crude protein (CP) and 29–35% fat and have an amino acid pattern comparable to fishmeal [35,36,37,38].

Insects are known to inhabit a wide variety of environments, including extreme ones, due to their adaptive behavioral and physiological mechanisms [39]. However, these tolerance mechanisms are not well understood [39]. Moreover, insects, along with other ectotherms, depend largely on ambient temperatures to regulate their metabolism and development rates [40]. Forecast modeling suggests that due to climate change, insects inhabiting more temperature-versatile geographic regions will survive elevated temperatures, while those inhabiting regions where little temperature variances occur will experience a decline in their populations as global warming proceeds [41,42]. BSF, originally traced back to the Americas, is currently known to be found in tropical, as well as temperate, regions across the globe [34]. Various studies have looked into the effects of different diets on laboratory-reared BSF [34,43,44,45,46], as well as the influence of temperature on the development and survival of BSF larvae using laboratory-prepared diets [47]. Other studies have investigated the influence of organic waste streams as rearing substrates on the development and survival of BSF larvae [25,48,49,50]. Yet, most of these studies were carried out with the aim of understanding and developing BSF larvae large-scale production systems in the developed world, where indoor climate-controlled facilities can be easily established. However, to the best of our knowledge, no study so far has investigated the combined influence of urban organic waste stream-based diets and temperature on the development and survival of BSF in the developing world context. 

Therefore, this study sought to investigate the influence of temperature on selected life-history traits of BSF reared on two different and readily available urban organic waste streams in the urban environment of a large city in SSA. This comparison allowed us to determine which of the two organic waste streams performs best, as well as the accompanying optimum temperatures. Information from this study is important for improving rearing methods in SSA, as well as for creating cost-effective and environmentally sustainable alternative livestock feeds that can buffer the impact of climate change, especially for small-scale livestock producers who are not connected to international feed markets and local feed producers who can neither afford nor implement sophisticated climate-controlled production facilities.

## 2. Materials and Methods

### 2.1. Study Location

The study was carried in the laboratories of the International Centre for Insect Physiology and Ecology (*icipe*), in Nairobi, Kenya.

### 2.2. Preparation of Substrates

The tested substrates, cow dung (CD) and brewers’ spent grain (SG), were both sourced locally. Fresh CD was collected from Farmers Choice slaughterhouse in Kahawa West in Nairobi; the bovines originated from different ranches in Kenya where they had been raised on natural grassland. SG was sourced from Tusker House, Kenya Breweries Ltd. off Thika Road in Nairobi after the fermentation of the barley in the beer production process. The substrates were chosen based on their availability in Nairobi with a view of their potential future use for large-scale industrial BSF larvae production. Fresh CD and SG substrates were oven dried at 60 °C for 48 h and then stored for subsequent experiments in a refrigerator at −20 °C.

### 2.3. Stock Colony

The stock population of BSF populations was maintained at the insectary in *icipe*. Adult BSF were housed in an outdoor, metal-framed cage with 1.5 mm screen mesh (1.8 × 1.8 × 1.8 m) with direct access to daylight to encourage mating. The flies were supplied with water to prolong their life. Corrugated cardboard and some SG were placed within the cage to attract adult females for oviposition. The colony was maintained in the insectary for over 8 generations before use in this experiment.

### 2.4. Experimental Set-Up

First, 10 batches of eggs were collected from the stock colony and placed into smaller containers (15 × 9.4 cm) containing an oviposition substrate of moist-to-liquefied SG (100 g). Each setup was closely monitored 3 times a day to ensure egg hatching. After hatching, 100 3–5-day-old larval instars were transferred into different, clear, plastic, 500-mL containers with the 2 test substrates, CD and SG. Each container contained 100 g of the test substrate, which was mixed with water to achieve a moisture content of 70% by weight [51]. The experiment was conducted in incubators (MIR-554-PE, Sanyo/Panasonic cooled incubators, Osaka, Japan) with air humidity of 70% and a photoperiod of 12L: 12D. Each substrate was subjected to different temperature treatments of 15 °C, 20 °C, 25 °C, 30 °C, and 35 °C. Each substrate–temperature treatment was replicated 5 times and was aerated daily to ensure that the substrate was thoroughly turned and well moisturized.

Each treatment was monitored daily, and the duration of the larval development was recorded. The recording of larval development stopped when all the larvae reached the prepupal stage. The prepupae were removed, weighed, and placed individually in separate, labeled, 35-mL plastic cups filled with moist sawdust. Each cup with the prepupae was covered with a breathable lid and returned to its respective temperature regime for daily monitoring and subsequently recorded for the numbers of puparia formed. Further, adult emergence was monitored daily. Upon emergence, 10 2-day-old adults (5 males and 5 females) from every replicate per substrate–temperature treatment were transferred into a cage (40 × 40 × 40 cm) and allowed to mate for 24 h at their respective temperatures. Thereafter, an oviposition device with a small bowl of moist chicken manure and 2–3 cardboards were placed in each cage to provide sites for oviposition. A 10% sugar solution in water was provided daily in a vial through a filter paper inserted into the vial’s lid. The laid egg batches were recorded, and the numbers of eggs per batch were counted under a microscope. The adult flies’ longevity was recorded daily until all the caged flies were dead. 

### 2.5. Statistical Analysis

R Statistics (version R 3.3.3, R Foundation for statistical computing, Vienna, Australia) and Stata Statistical Software (Release 15.1, StataCorp, College Station, TX, USA) were used for analyses. The collected data were subjected to Levene’s test for normality, followed by a two-way analysis of variance (ANOVA) using the general linear model (GLM) procedure. Where significant differences existed, Tukey Honest Significant Difference (HSD) or Least Significant Difference (LSD) post hoc was used to separate the means at the *p* < 0.05 level. For temperature-driven models, a parametrized square function [52] was fitted to the developmental time stage-specific data of the insect. The linear model expressed below evaluated the relationship between BSF developmental times and temperatures [51]:

*y* = *a*(*x* − *b*)^2^ + *c* (1)

where *b* is the temperature for the minimum development time and *c* is the minimum development time.

## 3. Results

### 3.1. Development of BSF Larvae

Both temperature and substrate type significantly influenced BSF larval development, with the SG-fed BSF larvae needing significantly less time to reach prepupal stage in the temperature treatments tested. The time needed for larval development decreased gradually with the increasing temperatures and was the shortest at 30 °C for the SG-fed larvae and 35 °C for the CD-fed larvae (Table 1). The CD-fed BSF needed 24%, 63%, 64%, and 65% less time at 20 °C, 25 °C, 30 °C, and 35 °C, respectively, in comparison with the time needed at 15 °C. Similarly, the SG-fed BSF needed 53%, 84%, 88%, and 86% less time at 20 °C, 25 °C, 30 °C, and 35 °C, respectively, in comparison with the time needed at 15 °C.

Prepupal weight was significantly influenced by both temperature and substrate type, with the SG-fed BSF larvae weighing more than those fed with CD. Prepupal weights increased with the increasing temperatures, with the prepupae reared on CD substrate weighing the most at 30 °C, while those reared on SG were heaviest when reared at 25 °C and 30 °C (Table 2). At their heaviest (30 °C), the weight of the CD-fed prepupae increased by 33%, while that of the SG-fed prepupae increased by 20% in comparison with their lightest weight (15 °C). On the other hand, the weight of the SG-fed prepupae was 51%, 38%, and 46% greater than that of the CD-fed prepupae at 15 °C, 30 °C, and 35 °C, respectively.

### 3.2. Development of BSF Pupae

Pupal developmental time differed significantly across different temperatures for BSF pupae previously reared on both substrates. The pupal developmental time decreased gradually with increasing temperatures and was shortest at 35 °C and 30 °C for prepupae reared on CD and SG substrates, respectively (Table 3). Pupae reared on SG needed significantly less time to emerge as adults than those reared on CD. For instance, pupae reared on CD needed 28%, 70%, 72%, and 74% less time at 20 °C, 25 °C, 30 °C, and 35 °C, respectively, in comparison with the time needed at 15 °C. Similarly, pupae reared on SG needed 59%, 85%, 95%, and 91% less time, respectively, in comparison with the time needed at 15 °C. Moreover, pupae reared on SG needed 22%, 53%, 59%, 75%, and 64% less time than the ones reared on CD at 15 °C, 30 °C, and 35 °C, respectively.

### 3.3. Longevity and Fecundity of BSF Adults

The longevity of BSF adult flies was significantly influenced by both temperature and substrate type, with BSF adults previously reared as larvae on SG living significantly longer than those previously reared on CD (Table 4). Longevity decreased with increasing temperatures, with all BSF adults irrespective of their larval rearing substrate living the longest at 15 °C. For instance, the longevity of BSF adults previously reared on CD at 20 °C, 25 °C, 30 °C, and 35 °C decreased by 26%, 28%, 37%, and 46%, respectively, in comparison with longevity at 15 °C. Similarly, the longevity of BSF adults previously reared as larvae on SG decreased by 11%, 28%, 35%, and 48%, respectively, in comparison with longevity at 15 °C.

Both temperature and substrate type significant affected the number of eggs laid or oviposited by adult BSF (Table 5). BSF adults obtained from larvae previously reared on both CD and SG produced more eggs at higher temperatures and the most eggs at 30 °C. However, at 35 °C, egg production by adult BSF previously reared on both CD and SG declined by 27% and 39%, respectively. The eggs produced by flies derived from the SG substrate produced 34% more eggs than those derived from the CD substrate at 30 °C.

### 3.4. A Linear Temperature-Driven Model (Square Function)

**Figure 1:**
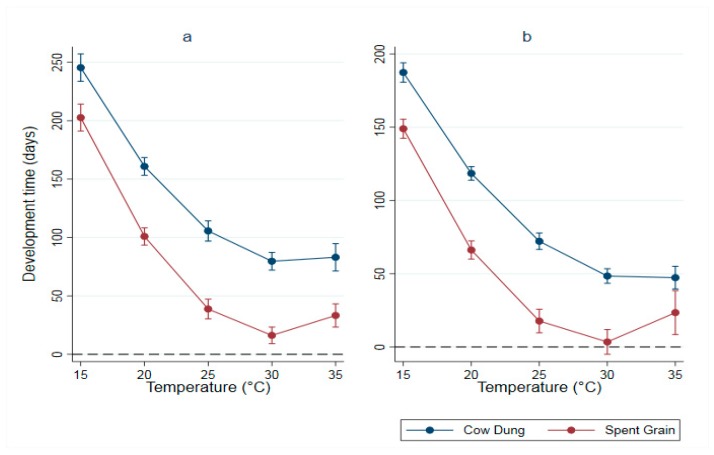
Temperature-driven model (parametrized square function) of (**a**) larval and (**b**) pupal developmental time (in days) of black soldier fly reared on two different organic substrates at five different temperature regimes.

The mean minimum development duration was estimated to be 77.5 days (range 69.5–85.5 days) at 31.9 °C (range 30.2–33.7 °C) for CD-fed immatures and 16.1 days (range 9.0–23.2 days) at 30.3 °C (range 29.4–31.3 °C) for SG-fed ones (Figure 1a, Table 6). For the pupae, the mean minimum development time was estimated to be 45 days (range 39.4–50.6 days) at 32.8 °C (range 39.1–50.9 °C) (Figure 1b, Table 7). The mean temperature threshold for SG-fed pupae was not significant.

## 4. Discussion

Temperature has proven to be a key factor in the development and survival of insects [53]. Moreover, it is well established that BSF larvae are sensitive to their external environments and that temperatures influence their development and survival [47,54]. On the other hand, temperature and nutrition interact to affect key life-history traits in insects, such as maturity, development rate, reproduction, and survival [55]. Several studies looked into the influence of either laboratory-reared diets at a constant temperature or organic side streams as feeding substrates on life-history traits of BSF larvae [44,45,46,47,56]. However, no previous study investigated the combined influence of temperature and urban organic waste material as rearing substrates in a developing world context. Those waste streams, cow dung and spent grain, were readily available in Nairobi, Kenya, and are arguably also available in other megacities in the developing world. We measured the influence of five different temperatures and two organic waste streams on the fitness of BSF larvae as a proposed alternative protein source for livestock feed. We measured the duration of development of immature BSF larvae, as well as BSF prepupae weights. We recorded significantly faster durations for BSF larvae and heavier weights for BSF prepupae reared on SG compared with those reared on CD even at the low temperatures of 15 °C and 20 °C. The development times of BSF immatures reared on both substrates decreased with increasing temperatures. The weights of BSF prepupae increased with increasing temperatures and were the heaviest at 25 °C and 30 °C. 

Several factors may have contributed to the differential development observed between the two rearing substrates. The most important contributing factor was the difference in the quality of the nutritional content between the rearing substrates. Several studies emphasized the importance of nutritional components, such as proteins and carbohydrates, in the development of insect larvae [57,58,59]. Therefore, we assume that SG better provided the BSF immatures with the nutritional resources and energy required to complete their development stage faster. This observation is supported by findings of Harnden and Tomberlin [60], who recorded faster development for BSF larvae reared on a grain-based diet in comparison with those reared on an animal tissue diet at 32.2 °C. Meneguze et al. [27] also reared BSF larvae on SG but recorded faster durations in comparison with what we report in this study. Yet, on the other hand, we noted heavier weights for BSF prepupae reared on SG than Tomberlin et al. [43] in a similar study. The main reasons for these discrepancies are differences in methodologies and experimental set-ups, as well as varying temperatures at which the BSF larvae were kept. Another factor that may have influenced the overall development of BSF could be related to its phenotypic plasticity. Phenotypic plasticity is the ability of an individual organism to alter its phenotype or to modify developmental events in response to changes in environmental conditions, allowing it to maintain high fitness regardless of the environmental variability [61,62]. Phenotypic plasticity permits organismal diversification within species without having to couple it with speciation through the evolution of environment-specific responses in phenotype expression [62]. The stock colony from which we obtained the BSF eggs was housed in an outdoor insectarium subjected to light cycles and temperature regimes reflective of the seasonality in Nairobi. Zhou et al. [63] collected BSF strains from three different climatic regions in the USA and China, reared them under identical conditions, and showed that they could reveal strikingly different BSF life-history traits. They attributed such differential development to the phenotypic plasticity of BSF. Further studies are needed to verify whether phenotypic plasticity in BSF is exclusively influenced by the environment or may also be genetically determined. 

Food availability and access to nutritional resources are other crucial factors affecting larval and adult life history traits [64,65]. For instance, the weight of BSF prepupae reared on CD in our study were lighter than those recorded by Myers et al. [56] for prepupae reared on a similar type of substrate. While Myers et al. [56] provided the larvae with fresh increments of CD on a daily basis, we opted for a lump sum amount of CD at the start of our experiment. Unlike fresh incremental diets, materials in lump sum diets age with time, leading to reduced amounts of nutritional components, such as proteins and carbohydrates, which are crucial for the development of insect larvae [57,58,59]. Facing such reductions in nutritional components, larvae refer to compensatory feeding, leading to faster development times and reduced weight gains [66,67]. This is also corroborated by Sheppard [68], who observed an optimal development of BSF reared on fresh CD provided at low increments. The consistency and physical texture of the rearing substrates used in our study may also have affected the results. Although, we did not specifically test the consistency and physical texture of the rearing substrates, it was visually evident during our experiments that CD was quite thick in texture and therefore may have limited the BSF immatures’ mobility and access to the little amount of nutrients available, consequently affecting their life-history traits. Most importantly, the chemical composition of cow dung has been extensively summarized by Azevedo and Stout [69] and Graber [70], showing a high fiber ratio of about 27% and a proportionately lower percentage of protein. A complex set of factors influence the extent to which fiber will be digested by BSF, including the physical state of the cow dung, the level of intake, and the amount of readily fermented nutrients (i.e., carbohydrate and protein) in the ration. Moreover, cow dung constituent of largely non-nutritive elements and the variability of BSF ability to break down fiber might explain the considerable variation observed using the two substrates regardless of the rearing temperature. On the other hand, brewer’s spent grain has been found to contain several essential nutrients, which are crucial for BSF growth. Couch [71] reported a proximate constituent of over 20% crude protein, about 6% ether extract, over 15% crude fiber, and 4% ash in brewer’s spent grain. This is further supported by the National Research Council NRC [72], which reported that spent grain contains 25.3% crude protein (CP), 6.3% crude fat, and around 2080 Kcal/Kg of metabolizable energy and that spent grain is also a good source of B vitamins, thus rendering it a good potential substrate in BSF production. The use of spent grain in BSF diet compared with cow dung might be the reason for the improvement in the body weight gain of BSF prepupae, which translates to an increased profit margin. We did not conduct any tests on the influence of temperature and diet on food ingestion or substrate reduction, as our objective was to test the influence of temperature and diet on the development of BSF in terms of development duration, weight, longevity, and fecundity. However, based on our visual observations, BSF consumed SG more efficiently than CD, indicating that waste reduction might also be influenced by the nutritional quality, texture, and moisture content of the substrate.

Pupation, a complex process involving significant morphological and physiological transformations, is essential for holometabolous insects [73]. Therefore, we additionally measured the duration of pupation, as well as the adult longevity and fecundity, as affected by the previously experienced temperature and substrate regimes. Adult emergence took longer at lower temperatures and was significantly shortest at 25–35 °C and shorter for BSF previously reared on SG than those reared on CD at those temperatures. The relationship between temperature and adult emergence observed in our study is not uncommon in insects. For instance, Telles-Romero et al. [73] studied the effects of four temperature regimes (18 °C, 20 °C, 25 °C, and 30 °C) on the West Indian fruit fly *Anastrepha obliqua* and found a decrease in the duration until adult emergence with increasing temperatures. Moreover, moist sawdust, the pupation substrate used in our study, may have also collectively accelerated the developmental time of pupae to adult emergence. Our results are further supported by Holmes et al. [48], who also observed low pupal mortality, a higher proportion of adult emergence, and increased adult longevity when using wood shavings and concluded that such a pupation substrate significantly enhances BSF development. The reason for this is most likely the high moisture content (70%) and low compaction density of wood shavings, which facilitates pupation and the emergence of BSF [48].

Adult longevity significantly decreased with increasing temperatures, with BSF adults derived from larvae previously reared on SG recording higher longevity. This confirms our previously stated assumption regarding the influence of the nutritional content of the rearing substrate, as well as access to nutritional resources, on larval and adult life-history traits and explains why we observed –even at a similar temperature range (25–30 °C)—shorter adult longevity in comparison with Myers et al. [56], who also reared BSF on CD. However, they noted greater longevity in adults that were previously fed with higher increments of fresh CD as BSF larvae. Moreover, because adult BSF do not feed and only consume water, exposing them to high temperatures will cause dehydration leading to an increased mortality rate and reduced lifespan [44]. BSF fecundity was highest at 30 °C and was significantly affected by the type of substrate fed to the larvae. The higher weight gain recorded at the prepupal stage is likely to translate into larger adult body size in both males and females [74]. Several studies have reported that larger-sized females lay more eggs due to their greater energy reserves [74]. Although we did not measure the size of the BSF females, the fact that the females that emerged from larvae reared on SG had significantly higher fecundity compared with those from CD, could clearly point in this direction. 

## 5. Conclusions and Outlook

Our study is the first to provide information regarding the influence of temperature on the life history of BSF reared on two diets readily available in urban SSA. Such information is necessary for developing BSF potential in the developing world, both as a tool for the bioconversion of organic waste and as an alternative protein source in feed stock. Our results demonstrate that both temperature and substrate type significantly influence the development, longevity, and fecundity of the flies. Black soldier flies needed less time to develop on higher temperatures during their immature and pupal stages, while adults’ longevity decreased at higher temperatures. Similarly, BSF reared on spent grain outperformed the ones reared on cow dung by surpassing them in weight and requiring less time to develop. Regardless of the waste stream used, BSF production systems have to be designed in a manner that provides BSF with adequate access to fresh nutritional content. Also, considering that the BSF could be used to feed livestock that are part of the human food chain, it is important to assess the potential risks associated with contamination by pathogens and the bioaccumulation of heavy metals. Moreover, the influence of the rearing substrate influenced the fecundity of adult flies, with the ones reared on spent grain as larvae producing more eggs, underlining the importance of the nutritional quality of the rearing substrate. Considering that BSF is produced for feed and not only for the purpose of organic waste recycling, this interesting information suggests the need to introduce starter-culture production facilities with customized rearing substrates for the production of BSF eggs as compared with organic waste recycling facilities. Hence, future research should focus on the development of adapted technologies in terms of the following: (1) rearing temperatures, (2) feeding methods, and (3) substrate hygiene and safety measures for small- to medium-scale industrial mass production systems of insects, such as BSF, into which commonly available urban organic waste streams can be fed. The availability of such production systems would considerably lower the cost of livestock feeds and consequently would make animal protein more affordable for the growing urban populations in SSA, thereby improving food security and nutrition, especially for women, children, and other vulnerable members of society.

## Figures and Tables

**Table 1:** Mean (±SD) duration of development (in days) of black soldier fly larvae reared on two different organic substrates at five different temperature regimes.

Temperature (°C)	CD-fed BSF	SG-fed BSF
15	238.800 ± 0.450 ^a^A	206.200 ± 5.020 ^b^A
20	180.400 ± 32.810 ^a^A	96.300 ± 1.510 ^b^B
25	86.800 ± 2.170 ^a^B	32.800 ± 2.060 ^b^C
30	85.200 ± 3.960 ^a^B	24.700 ± 3.590 ^b^C
35	83.400 ± 4.040 ^a^B	29.600 ± 1.400 ^b^C

Means (n = 5) in the same row followed by different lowercase letters and in the same column by different uppercase letters are significantly different at *p* < 0.05; BSF, black soldier fly; CD, cow dung; SG, spent grain.

**Table 2:** Mean (±SD) prepupal weight (in grams) of black soldier fly reared on two different organic substrates at five different temperature regimes.

Temperature (°C)	CD fed BSF	SG fed BSF
15	0.076 ± 0.005 ^a^B	0.128 ± 0.004 ^b^B
20	0.082 ± 0.004 ^a^B	0.132 ± 0.010 ^b^B
25	0.088 ± 0.004 ^a^B	0.153 ±0.013 ^b^A
30	0.106 ± 0.027 ^a^A	0.156 ± 0.011 ^b^A
35	0.086 ± 0.005 ^a^B	0.137 ± 0.013 ^b^B

Means (n = 5) in the same row followed by different lowercase letters and in the same column by different uppercase letters are significantly different at *p* < 0.05; BSF, black soldier fly; CD, cow dung; SG, spent grain.

**Table 3:** Mean (±SD) pupal developmental time (in days) of black soldier fly reared on two different organic substrates at five different temperature regimes.

Temperature (°C)	CD-fed BSF	SG-fed BSF
15	182.909 ± 29.680 ^a^A	150.333 ± 32.260 ^b^A
20	131.090 ± 36.670 ^a^A	61.500 ± 16.240 ^b^B
25	54.428 ± 15.140 ^a^B	22.333 ± 3.810 ^b^C
30	51.820 ± 17.690 ^a^B	13.000 ± 2.110 ^b^C
35	48.256 ± 15.490 ^a^B	17.500 ± 3.690 ^b^C

Means (n = 5) in the same row followed by different lowercase letters and in the same column by different uppercase letters are significantly different at *p* < 0.05; BSF, black soldier fly; CD, cow dung; SG, spent grain.

**Table 4:** Mean (±SD) longevity (in days) of adult black soldier fly reared on two different organic substrates at five different temperature regimes.

Temperature (°C)	CD-fed BSF	SG-fed BSF
15	13.200 ± 1.304 ^a^A	14.200 ± 0.447 ^b^A
20	9.800 ± 2.683 ^a^B	12.600 ± 2.503 ^b^B
25	9.600 ± 0.548 ^a^B	10.200 ± 0.616 ^b^C
30	8.375 ± 0.518 ^a^B	9.200 ± 1.373 ^b^D
35	7.100 ± 0.316 ^a^C	7.400 ± 0.699 ^b^E

Means (n = 5) in the same row followed by different lowercase letters and in the same column by different uppercase letters are significantly different at *p* < 0.05; BSF, black soldier fly; CD, cow dung; SG, spent grain.

**Table 5:** Mean (±SD) number of eggs produced by black soldier flies reared on two different organic substrates at five different temperature regimes.

Temperature (°C)	CD-fed BSF	SG-fed BSF
15	0.000 ^a^E	238.800 ± 50.500 ^b^D
20	169.900 ± 90.750 ^a^D	422.000 ± 4.240 ^b^C
25	472.900 ± 79.560 ^a^C	503.800 ± 68.028 ^b^C
30	916.100 ± 125.110 ^a^A	1,230.400 ± 242.510 ^b^A
35	669.100 ± 25.260 ^a^B	751.800 ± 114.960 ^b^B

Means (n = 5) in the same row followed by different lowercase letters and in the same column by different uppercase letters are significantly different at *p* <0.05; BSF, black soldier fly; CD, cow dung; SG, spent grain.

**Table 6:** Estimates of linear model parameters describing the relationship between temperature and developmental time of black soldier fly larvae reared on two different organic substrates.

	CD fed BSF			SG fed BSF		
Model Parameter	Estimate (±SE)	*p*-Value	95% CI	Estimate (±SE)	*p*-Value	95% CI
a	0.586 ± 0.068	<0.001	0.445–0.723	0.792 ± 0.065	<0.001	0.661–0.923
b	31.925 ± 0.871	<0.001	30.174–33.676	30.348 ± 0.477	<0.001	29.389–31.307
c	77.490 ± 3.972	<0.001	69.504–85.477	16.114 ± 3.546	<0.001	8.983–23.244
R2	0.798			0.852		

Development time = a × (temperature − b) 2 + c; where b is the temperature for the minimum development time and c is the development time at the minimum development time; means (n = 5) (±SE) are significantly different at *p* < 0.05; CI = Confidence Intervals; BSF = black soldier fly; CD = cow dung; SG = spent grain.

**Table 7:** Estimates of linear model parameters describing the relationship between temperature and pupal developmental time of black soldier fly reared on two different organic substrates.

	CD fed BSF			SG fed BSF		
Model Parameter	Estimate (±SE)	*p*-Value	95% CI	Estimate (±SE)	*p*-Value	95% CI
a	0.452 ± 10.520	<0.001	0.367–0.536	0.685 ± 0.061	<0.001	0.566–0.804
b	32.751 ± 0.801	<0.001	31.164–34.337	29.581 ± 0.592	<0.001	28.416–30.746
c	45.014 ± 2.843	<0.001	39.423–50.606	n.s.	0.433	n.s.
R2	0.913			0.99		

Development time = a × (temperature − b) ^2^ + c; where b is the temperature for the minimum development time and c is the development time at the minimum development time; means (n = 5) (±SE) are significantly different at *p* < 0.05; CI = Confidence Intervals; BSF = black soldier fly; CD = cow dung; SG = spent grain; n.s, not significant.

## Data Availability

All relevant data are within the paper.
